# Reevaluating Metabolism in Alzheimer's Disease from the Perspective of the Astrocyte-Neuron Lactate Shuttle Model

**DOI:** 10.1155/2013/234572

**Published:** 2013-04-23

**Authors:** Jordan T. Newington, Richard A. Harris, Robert C. Cumming

**Affiliations:** Department of Biology, The University of Western Ontario, London, ON, Canada N6A 5B7

## Abstract

The conventional view of central nervous system (CNS) metabolism is based on the assumption that glucose is the main fuel source for active neurons and is processed in an oxidative manner. However, since the early 1990s research has challenged the idea that the energy needs of nerve cells are met exclusively by glucose and oxidative metabolism. This alternative view of glucose utilization contends that astrocytes metabolize glucose to lactate, which is then released and taken up by nearby neurons and used as a fuel source, commonly known as the astrocyte-neuron lactate shuttle (ANLS) model. Once thought of as a waste metabolite, lactate has emerged as a central player in the maintenance of neuronal function and long-term memory. Decreased neuronal metabolism has traditionally been viewed as a hallmark feature of Alzheimer's disease (AD). However, a more complex picture of CNS metabolism is emerging that may provide valuable insight into the pathophysiological changes that occur during AD and other neurodegenerative diseases. This review will examine the ANLS model and present recent evidence highlighting the critical role that lactate plays in neuronal survival and memory. Moreover, the role of glucose and lactate metabolism in AD will be re-evaluated from the perspective of the ANLS.

## 1. Introduction

The human brain consumes approximately 20% of the body's total energy yet only represents 2% of the total body mass, far outweighing the demand of other organs in the body. While other tissues in the body rely on a variety of energy sources, the brain is believed to primarily depend upon the oxidation of glucose to meet its energy demands. The majority of the energy produced by the oxidation of glucose is used for the maintenance and restoration of ion gradients associated with synaptic transmission, as well as uptake and recycling of neurotransmitters [1]. As an essential organ, the brain requires adequate glucose and oxygen delivery from the vasculature system, a process controlled by the precise regulation of energy supply and demand. Consequently, changes in brain activity are accompanied by changes in cerebral blood flow, a phenomenon which forms the basis of functional brain imaging technologies. For decades, glucose has been considered as the main, if not exclusive energy substrate for the adult brain. Glucose is normally metabolized through the glycolytic pathway to pyruvate and, in the presence of oxygen, is fully oxidized to CO_2_ and water in the mitochondria. Over 17 times more energy is produced from mitochondrial respiration than from glycolysis (34 adenosine triphosphate (ATP) versus 2, respectively). Therefore, neurometabolism has traditionally been perceived as a process with a strict reliance on the oxidation of pyruvate in the mitochondria in order to meet the high energy needs of neurons.

Aerobic glycolysis, also known as the Warburg effect, is defined as glucose utilization in excess of that used for mitochondrial respiration despite sufficient oxygen to completely oxidize glucose for maximal ATP generation. A by-product of aerobic glycolysis is lactate, a metabolite which is normally exported out of cells. Lactate has traditionally been perceived as a dead-end product of glycolysis under hypoxic conditions, most commonly produced by skeletal muscle during exercise. However, in 1985 Brooks proposed that lactate produced by skeletal muscle during exercise is shuttled through the interstitium and vasculature to other sites in the body where it can be used as an oxidative metabolite [2]. Despite evidence suggesting that lactate is a valuable fuel source in the body, its presence in the brain has been interpreted as a sign of cerebral harm. Though lactate has long been considered a potentially toxic metabolic waste product, it is now recognized as not only a valuable energy substrate for CNS neurons but even as a preferred source of energy under certain circumstances [3, 4].

Over the last few decades, key information about brain metabolism has been gathered using positron emission tomography (PET) imaging. PET allows for the *in vivo* determination of the cerebral metabolic rate of glucose consumption, cerebral metabolic rate of oxygen consumption, and cerebral blood flow. Traditionally, ^18^F-labeled fluorodeoxyglucose- (FDG-) PET signals were believed to primarily measure glucose utilization by neurons due to the high energy demand of this cell type during activation [5]. However, in the mid to late 1980s, an important series of PET studies challenged this assumption by showing that cerebral glucose consumption exceeds oxygen utilization in certain regions of the human brain [6, 7]. These early observations suggested that the metabolic needs of active neural tissue are met in a partially nonoxidative manner [6, 7]. More recently, Vaishnavi and colleagues, using a more refined PET analysis of 33 healthy adults, identified high rates of aerobic glycolysis in the medial and lateral parietal and prefrontal cortices, regions known to participate in cognitive control networks [8]. These observations brought support to the notion that the metabolic needs of active brain tissue are met, at least partially, by aerobic glycolysis. Further support was provided by various *in vivo* ^1^H-magnetic resonance spectroscopy (MRS) studies in healthy adults which showed activity-dependent increases in lactate levels (the end product of aerobic glycolysis) in brain areas similar to those found in the PET studies [9–11]. The question then arises as to whether the changes in glucose metabolism (oxidative versus glycolytic) are taking place in different cellular compartments of the brain and/or are dependent on the specific needs of these areas.

## 2. The Astrocyte-Neuron Lactate Shuttle (ANLS) Model

The conventional view of glucose metabolism in the brain contends that glucose is the principle substrate for oxidative metabolism in both neurons and astrocytes [12, 13]. From this perspective, activation of nerve cells results in increased Na^+^K^+^ATPase activity and ATP consumption, which leads to the activation of the glycolytic pathway in both neurons and astrocytes [14]. However, during nerve cell activation, transient increases in the rate of glycolysis, in excess of the rate of respiration, are believed to be responsible for the production of lactate, which can be produced by both neurons and astrocytes [13, 15]. The subsequent buildup of lactate is believed to be harmful; therefore, its clearance via the circulatory system or uptake by supporting cells is necessary following nerve cell activation [12, 15]. However, studies performed in the early 1990s have challenged this conventional view of nerve cell metabolism and led to the proposal that lactate itself may function as a primary fuel source for neurons [16–18].

In most regions of the human brain astrocytes outnumber neurons [19]. These specialized glial cells play a key role in numerous functions of the brain including modulation of synaptic ion and neurotransmitter levels, defense against oxidative stress, and regulation of synapse formation and remodeling [20]. Astrocytes possess a unique morphology and spatial distribution that enable them to provide energy substrates from capillaries to neurons. At the morphological level, astrocytes are stellate shaped and have two types of specialized processes. First, astrocytes contain processes called perivascular end-feet which cover cerebral blood vessel capillaries [20, 21]. Importantly, both endothelial and astrocytic cells express the glucose transporter GLUT1 at the surface of these structures to allow for the efficient transfer of glucose into the CNS [22]. Additionally, astrocytes have fine perisynaptic processes that wrap around synapses in a dynamic fashion [20, 21, 23]. These features position astrocytes as the prevalent site of glucose entry into the brain and endow them with the capacity to sense neuronal activity at the synapse and respond with the appropriate metabolic supply from the vascular system [24]. Another key feature of astrocytes is that they are the primary cell type within the brain that can store glucose as glycogen [25–27]. Glycogen represents the major energy reserve in the brain during periods of low glucose (aglycemia). During increased activity, glycogen is broken down to lactate (a process termed glycogenolysis) to fuel neuronal metabolism [25]. Brain cells can utilize various energy substrates besides glucose, including lactate, pyruvate, glutamate, and glutamine [28].

**Figure 1:**
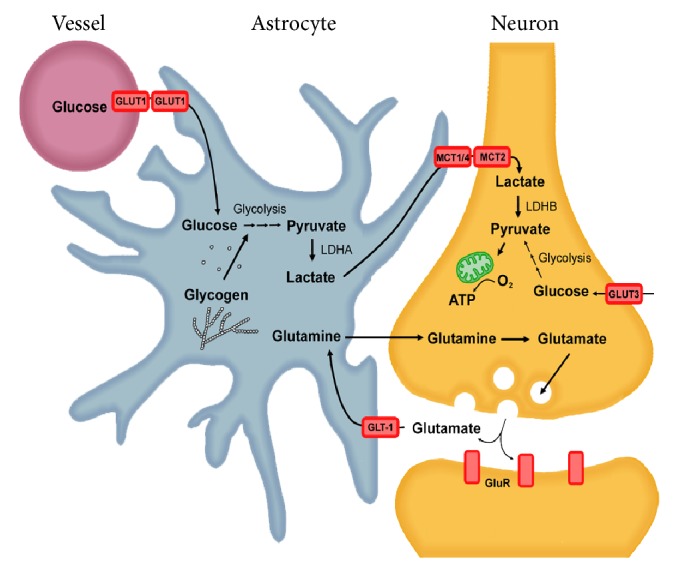
The astrocyte-neuron lactate shuttle hypothesis. The activation of nerve cells leads to the release of the neurotransmitter glutamate. Glutamate is actively taken up into astrocytes by glutamate transporters (GLT-1) and is converted into glutamine. The uptake of glutamate into astrocytes stimulates both increased glucose uptake from surrounding capillaries via glucose transporters (GLUT1) and increased aerobic glycolysis. Aerobic glycolysis can also be stimulated by the breakdown of intracellular stores of glycogen. Pyruvate is converted to lactate by lactate dehydrogenase isoenzyme A (LDHA) and is exported out of the cell by the monocarboxylate transporter 1 or 4 (MCT1/4) and transported into nerve cells via MCT2. LDHB within nerve cells coverts lactate to pyruvate which is used to fuel oxidative phosphorylation within mitochondria. Glucose can also enter nerve cells via GLUT3 transporters.

Among these substrates, lactate has garnered considerable attention recently as an important fuel source for the brain. Astrocytes readily take up glucose from cerebral blood vessels, via GLUT1, and process it at a high capacity by aerobic glycolysis to produce lactate which is released into the extracellular space via monocarboxylate transporters (MCTs). Extracellular lactate is then taken up by neurons and converted to pyruvate, likely by lactate dehydrogenase (LDH). Pyruvate is subsequently converted to acetyl CoA, through the activity of the pyruvate dehydrogenase complex and then enters the tricarboxylic acid (TCA) cycle followed by the generation of nicotinamide adenine dinucleotide (NADH) to fuel oxidative phosphorylation via the mitochondrial electron transport chain. In addition, astrocytes take up glutamate, the primary excitatory neurotransmitter in the CNS, and convert it to glutamine as part of a recycling mechanism. In doing so, glutamate triggers a cascade of molecular events leading to an enhancement of glucose utilization by astrocytes [16]. Collectively, these findings led to the formation of the astrocyte-neuron lactate shuttle (ANLS) model (Figure 1), first proposed 18 years ago, which posits that (1) neuronal activity increases extracellular glutamate which is taken up by astrocytes via glutamate transporters leading to (2) a triggering of glucose uptake and aerobic glycolysis in astrocytes which (3) leads to a large increase in the production of lactate followed by its release into the extracellular space and (4) transport into neurons where it is used as an energy substrate for oxidative and nonoxidative derived ATP production (for review see [29]). Both astrocytes and neurons have the capacity to fully oxidize glucose and/or lactate [28]. However, astrocytes and neurons preferentially use different metabolic pathways which is, in part, due to cell type-specific expression patterns of key genes regulating energy metabolism, as discussed below.

## 3. Evidence in Support of the ANLS Model

Glutamate is the primary excitatory neurotransmitter of the cerebral cortex. The ANLS model contends that astrocytic lactate production is tightly coupled to neuronal activation and glutamate release and subsequent glutamate uptake from the astrocytes. The first evidence for the existence of an ANLS was demonstrated by Pellerin and Magistretti in 1994, who showed that glutamate uptake in primary mouse cortical astrocytes stimulated aerobic glycolysis (glucose uptake and lactate production) [16]. Similar results were obtained with cultured glial Müller cells from the retina [30]. Moreover, using* in vivo* ^13^C-NMR data from the rat brain cortex researchers showed that the rate at which glucose is oxidized within neurons is equal to the rate of glutamate cycling over a range of EEG activity (from isoelectric up to near-resting levels) [31]. Furthermore, knockout or knockdown of glutamate transporters results in a decrease in glucose uptake and lactate release from astrocytes in activated areas, suggesting that the regulation of brain metabolism is tightly regulated by synaptic activity [23, 32]. Astrocytes express GLUT1 which is responsible for the uptake of glucose from associated capillaries. In contrast, neurons primarily express GLUT3 transporters. Interestingly, *Glut3* haploinsufficient (GLUT3+/−) mice exhibit similar rates and amounts of glucose uptake in the brain as wild-type mice [33]. In contrast, *Glut1* haploinsufficiency (GLUT1+/−) results in decreased glucose uptake and severe neurological defects, suggesting that essential glucose uptake in the CNS primarily occurs in a GLUT1-dependent manner in astrocytes [34]. Furthermore, neuronal activity triggered by whisker stimulation, increases glucose uptake in astrocytes but not in neurons [35]. Thus astrocytes appear to be responsible for providing nerve cells with metabolic substrates in an activity-dependant manner. However, it should be noted that GLUT3 is found in abundance in synaptic membranes and transports glucose up to 7 times faster than GLUT1 transporters [36, 37]. Moreover, glucose has been found to be evenly distributed throughout the brain. As a readily available substrate, glucose is likely to be used to varying degrees in different subsets of neurons throughout the brain [38]. The uptake of glucose or lactate by neurons is therefore dependent on the location and needs of the cell.

The observed production of lactate in astrocytes following glutamate exposure led researchers to hypothesize that lactate released by astrocytes may be taken up by adjacent nerve cells and utilized as a primary energy source [16]. The interconversion between lactate and pyruvate is catalyzed by the enzyme lactate dehydrogenase (LDH). Immunohistochemical analysis of postmortem tissue taken from the hippocampus and occipital cortex revealed a selective distribution of LDH isoforms including LDHA (muscle type) and LDHB (heart type) in astrocytes and neurons, respectively [17]. There are five known LDH isoenzymes, each composed of tetramers containing different ratios of LDHA or LDHB. LDHA preferentially catalyzes the reduction of pyruvate to lactate whereas LDHB favors the oxidation of lactate to pyruvate. Thus the enrichment of LDHB, and associated conversion of lactate to pyruvate, within nerve cells would suggest a role for lactate as a fuel source. Indeed, using ^13^C labeling of lactate and NMR spectroscopy, it was shown that lactate released by astrocytes is taken up by neurons and used to drive the TCA cycle, as evidenced by increased labelling of TCA cycle intermediates [38, 39]. Similar *in vivo* studies in mice and rats revealed that not only does lactate cross the blood-brain barrier, it is almost exclusively metabolized by neurons [40, 41]. Interestingly, intracellular delivery of glucose to astrocytes can sustain neuronal synaptic transmission during glucose deprivation [42]. However, this effect was suppressed in the presence of an inhibitor of MCT lactate transporters, supporting the hypothesis that glucose taken up by astrocytes is metabolized to lactate, which is then released and taken up by nerve cells to sustain synaptic activity [42].

The preference for lactate as a fuel source has been observed in: cortical neurons [43], chick sympathetic ganglia [44], rabbit vagus nerve [45], and human brain *in vivo* [46]. Thus, these results suggest that astrocytes primarily rely on aerobic glycolysis in the cell cytosol whereas nerve cells primarily use mitochondrial oxidative phosphorylation to support their energy demands. Further support for this theory comes from studies in which ATP levels were measured in cultured glial and nerve cells from rat hypothalamus and cerebellum following exposure to oligomycin, an inhibitor of the mitochondrial F_o_-F_1_ATP-synthase. Interestingly, oligomycin treatment induced a significant decrease in ATP levels in neurons but not in glial cells, indicating that ATP production in neurons is dependent on mitochondrial function whereas glial cells produce ATP in a mitochondrial independent manner [47]. Moreover, neurons exhibit increased ATP production following addition of exogenous lactate to the culture medium, whereas glial cells are unresponsive to exogenous lactate [47]. Collectively, these results indicate that glucose is taken up into astrocytes in a glutamate-dependent manner, converted to lactate, and exported to fuel neuronal energy needs. However, it should be noted that both astrocytes and neurons have the capacity to fully oxidize glucose and/or lactate during periods of limited substrate availability [28].

Despite evidence supporting the existence of an ANLS, it remains a controversial hypothesis. It has been argued that the use of *in vitro* models (i.e., cultured neurons, astrocytes, and brain slices), in addition to flawed experimental designs, have led to the incorrect interpretation that neurons preferentially utilize lactate over glucose [48]. Alternative viewpoints have been proposed in which glucose, and associated oxidative metabolism, provides the main fuel source for the brain under normal activating conditions in sedentary or modestly physically active subjects. In contrast, alternative substrates, including lactate, may substantially contribute to brain energetics when glucose supply is inadequate (during strenuous physical work, exercise, or hypoxia) or when glucose supply is inadequate (during hypoglycemia or intense brain activity). Thus, it can be argued that lactate usage as a fuel source is a result of opportunity (i.e., a glucose-sparing substrate), not of preference or necessity [48]. Opponents of the ANLS await for more *in vivo *evidence to support the assertion that the ANLS is a prominent form of metabolism in the brain.

## 4. The Intracellular Mitochondrial Lactate Shuttle

With recent evidence strongly supporting the ANLS model the question arises as to how lactate is being processed at the intracellular level in neurons. Intracellular lactate may follow two fates; it can either be converted into pyruvate in the cytosol or directly shuttled into the mitochondria for further oxidation. Early studies demonstrated that lactate oxidation can occur within the mitochondria of liver, heart, and skeletal muscle cells [49, 50]. The discovery of both mitochondrial localized lactate transporters and LDH provided further evidence that lactate can fuel mitochondrial metabolism [49, 51]. In contrast, other groups failed to show either mitochondrial LDH or lactate oxidation, and it was claimed that skeletal muscle mitochondria do not contain significant amounts of LDH activity [52, 53]. These contradictory findings have led to a vigorous debate about whether mitochondrial oxidation of lactate occurs in skeletal muscle. However, more recent studies suggest that a mitochondrial lactate oxidation complex does indeed exist in neurons. Primary cerebellar granule cells were shown to transport and metabolize L-lactate in mitochondria [54]. Mitochondrial oxidation of lactate to produce ATP was also shown in a human astrocytic cell line [55]. Treatment with oxamate, an inhibitor LDH, abolishes mitochondrial lactate consumption [55]. Immunohistochemical analyses of rat brain revealed that MCT1, MCT2, and LDH colocalize with the inner mitochondrial membrane marker cytochrome oxidase (COX) in cortical, hippocampal, and thalamic neurons [56]. Additionally, these enzymes coprecipitated with COX from isolated mitochondria [56]. The oxidation of lactate to pyruvate is accompanied by the reduction of NAD+ to NADH, an important reducing agent in the cell. Therefore, increased oxidation of lactate within the mitochondria may aid in the supply of NADH for the electron transport chain. Interestingly, electrophysiological measurements testing the effects of malonate and oxamate, two different LDH inhibitors, and the neuronal activator glutamate in rat hippocampal samples suggest that L-lactate, not pyruvate, is the end product of neuronal glycolysis* in vitro *[57]. These observations have prompted the hypothesis that lactate is the major product of cerebral glycolysis, whether aerobic or anaerobic, neuronal or astrocytic, under rest or during activation [58]. An additional layer of complexity arises from an intracellular-mitochondrial lactate shuttle and mitochondrial oxidation complexes present within neurons [56]. However, more functional evidence is needed to confirm this hypothesis *in vivo.*

## 5. Astrocytic Lactate Production and Memory

Lactate has long been considered a metabolic dead end, hence the ANLS model has been met with considerable skepticism [12, 14]. However, this viewpoint has changed in light of growing evidence indicating that lactate transport from astrocytes to neurons is essential for long-term memory [27, 59]. Memory is a process in which information is encoded, stored, and retrieved. Short-term memories involve the retention of information for a brief period of time and are dependent on posttranslational modifications of proteins [60, 61]. Long-term memories are formed after learning, retention, and consolidation which require the activation of signalling cascades that lead to gene activation, protein synthesis, and the growth of new synaptic connections [60, 61]. The cAMP response element binding protein (CREB) is a nuclear protein that modulates transcription and plays a central role in long-term memory following phosphorylation-dependent activation [60–62]. Not surprisingly, memory and learning are metabolically demanding processes, which appear in part to be dependent on glycogen metabolism [27, 63, 64]. Glycogen represents the major energy reserve in the brain and is stored exclusively in astrocytes, not neurons [25–27]. During periods of low glucose or increased activity glycogen is broken down to lactate to fuel neuronal metabolism [25]. A role for glycogenolysis in long-term memory formation was first observed by Gibbs and colleagues who found that intracerebral injection of 1,4-dideoxy-1,4-imino-D-arabinitol (DAB) (a glycogen phosphorylase inhibitor) in day-old chickens resulted in a dose-dependent inhibition of long-term memory [64]. Recently, a more intensive study investigating the importance of astrocytic glycogenolysis and long-term memory was performed by Suzuki et al. who examined learning and memory in rats using an inhibitory avoidance (IA) test. To test for the importance of glycogenolysis in hippocampal astrocytes rats were injected with DAB either 15 min before or immediately after IA training [63]. Training led to a significant increase in extracellular lactate in the hippocampus which was abolished by DAB administration [27, 63]. DAB had no effect on short-term memory (tested an hour after) but blocked long-term memory (tested at 24 hr) [63]. Importantly, L-lactate coadministered with DAB rescued memory loss [63]. Similar results were obtained testing spatial working memory in rats using spontaneous alteration tasks [27]. Furthermore, astrocytic glycogenolysis also appears to be required for phosphorylation of CREB (pCREB), a key molecular event linked to memory formation [63]. DAB-induced reduction of pCREB activation was also rescued by exogenous L-lactate suggesting a possible signalling role for lactate [20, 63].

The ability to shuttle (uptake and release) lactate to various regions of the brain is dependent on MCT activity. Examination of both MCT mRNA and protein levels in mouse cortical tissues revealed that MCT1 and MCT4 were expressed almost exclusively in astrocytes, whereas MCT2 was strongly expressed in neurons [65–67]. Based on these findings Suzuki and colleagues demonstrated the importance of CNS lactate transport on memory by using intrahippocampal injections of antisense oligodeoxynucleotides to individually decrease expression of MCT1, MCT2, and MCT4 [63]. Decreased expression of MCT1 or MCT4 in astrocytes resulted in disrupted long-term memory formation that was rescued by exogenous administration of lactate but not glucose [63]. Disrupting the expression of neuronal MCT2 also resulted in loss of long-term memory which was not rescued by exogenous lactate or glucose, indicating that transport of lactate into neurons is required for long-term memory formation [63]. Taken together, these results suggest that the astrocytic lactate export by MCT-1 and/or MCT-4, and subsequent import into neurons through MCT2, is essential for long-term memory [63]. Moreover, these findings lend additional support to the ANLS model. However, the role of lactate transport in memory loss associated with neurodegenerative diseases has remained largely unexplored.

## 6. Altered Mitochondrial Metabolism and ROS Production in Alzheimer's Disease

Alzheimer's disease (AD) is one of the most common neurodegenerative disorders in the elderly characterized by a range of progressive cognitive deficits and memory loss. AD is strongly associated with widespread nerve cell death and the accumulation of extracellular plaques and intracellular neurofibrillary tangles within the brain [68, 69]. These plaques are primarily composed of amyloid-*β*-peptide (A*β*), a 39–42 amino acid peptide derived from the proteolytic cleavage of the amyloid precursor protein (APP) by secretases, including Presenilin 1 (Psen1), Presenilin2 (Psen2), and *γ*-secretase [68, 70, 71]. The amyloid cascade hypothesis, first proposed over 20 years ago, suggests that A*β* deposition in the brain is a primary causative agent of AD [72]. A*β*-induced neuronal toxicity is linked to mitochondrial dysfunction and increased reactive oxygen species (ROS) production [70–78]. The impairment of mitochondrial metabolism in AD has been well documented [79, 80]. It is not entirely clear how A*β* perturbs mitochondrial function; however, A*β* has been shown to accumulate directly within mitochondria of CNS neurons in AD patients and in transgenic mice [80–83]. Moreover, A*β* has been shown to directly interact with and inhibit the enzymatic activity of alcohol dehydrogenase (ABAD) in mitochondria [82]. Disrupting the interaction between A*β* and ABAD reduces mitochondrial derived ROS and increases oxygen consumption and mitochondrial activity [84]. A*β* has also been shown to bind directly to cytochrome oxidase (COX) subunit 1, a member of complex IV of the electron transport chain, which likely accounts for the decreased activity of this enzyme in AD [85]. Additionally, treatment of SK-N-SH cells (a human neuroblastoma cell line) with A*β* results in a dose-dependent decrease in mRNA levels of mitochondrial COX subunits [86]. Human mitochondrial transcription factor-1 (TFAM) mRNA levels are also decreased following A*β*-treatment [86]. Moreover, reduced activity of enzymes participating in the TCA cycle and electron transport chain have been well documented in the brains of patients with AD [87–89]. In summary, A*β*-mediated perturbation of mitochondrial electron transport and metabolism results in increased ROS production and damage to essential cellular components such as nucleic acids (DNA/RNA), lipids, and proteins in the brain.

## 7. Activation of Aerobic Glycolysis Promotes Resistance to A***β***

Interestingly, nerve cells selected for resistance against A*β* toxicity exhibit a shift in metabolism to favor lactate production [90]. These changes are linked to increased glucose uptake and flux through the glycolytic pathway [91]. This switch in metabolism from mitochondrial respiration to aerobic glycolysis is a common phenotype of cancer cells and is driven in part by the hypoxia-inducible factor 1*α* subunit (HIF-1*α*) [92]. HIF-1*α* is a heterodimeric transcription factor that is stabilized in low oxygen environments (hypoxia) to facilitate metabolic adaptation to hypoxic conditions. HIF-1*α* activation induces the transcription of glucose transporters, glycolytic enzymes, and LDHA, thereby increasing the conversion of glucose to lactate [93, 94]. Moreover, HIF-1*α* plays a role in shutting down mitochondrial respiration by upregulating the expression of pyruvate dehydrogenase kinase 1 (PDK1) [95]. PDK1 phosphorylates and inhibits pyruvate dehydrogenase complex (PDH), limiting the oxidation of pyruvate within the mitochondria and decreasing oxygen consumption [92]. In addition to maintaining energy homeostasis in low oxygen environments HIF-1*α* stabilization results in a decrease in ROS production [95]. Indeed, HIF-1*α* stabilization in cancer cells results in decreased respiration and associated ROS production which renders these cells more resistant to apoptotic stimuli [96].

Similar to cancer cells, the stabilization of HIF-1*α* accounts for the observed metabolic changes in A*β*-resistant cells [91]. Interestingly, A*β*-resistant nerve cells also express increased levels of PDK1 accompanied by increased LDHA activity and lactate production when compared to control cells [90]. As a result, mitochondrial derived ROS, which is closely associated with A*β* toxicity, is markedly diminished in resistant relative to sensitive cells [90]. Chemical or genetic inhibition of LDHA or PDK1 resensitizes resistant cells to A*β*-induced cell death [90]. Moreover, overexpression of either PDK1 or LDHA in a rat CNS cell line confers resistance to A*β* and other neurotoxins, which is associated with a lower mitochondrial membrane potential and decreased ROS production [97]. PDK1- and LDHA-overexpressing cells also exhibit decreased oxygen consumption but maintain levels of ATP under both normal culture conditions and following A*β* treatment [97]. Interestingly, PDK1- and LDHA-overexpressing nerve cells are still sensitive to mitochondrial inhibitors such as rotenone, antimycin, and oligomycin [97]. These findings indicate that although activation of aerobic glycolysis confers resistance to A*β*, cells which adopt this metabolism still require a functional mitochondrial electron transport chain (ETC). The significance of this paradoxical observation is unknown but may indicate that a functional ETC is required for efficient mitochondrial oxidation of lactate and ATP production. Moreover, recent evidence showed supplementation with the oxidative energy substrates pyruvate and 3-beta-hydroxybutyrate *in vitro* or *in vivo* decreased A*β*-induced neuronal dysfunction supporting a protective role of active ETC in AD [98]. However, it is uncertain how these oxidative energy substrates restore neuronal function, and their effects on mitochondrial derived ROS in cell culture and animal models of AD remain unknown.

**Figure 2:**
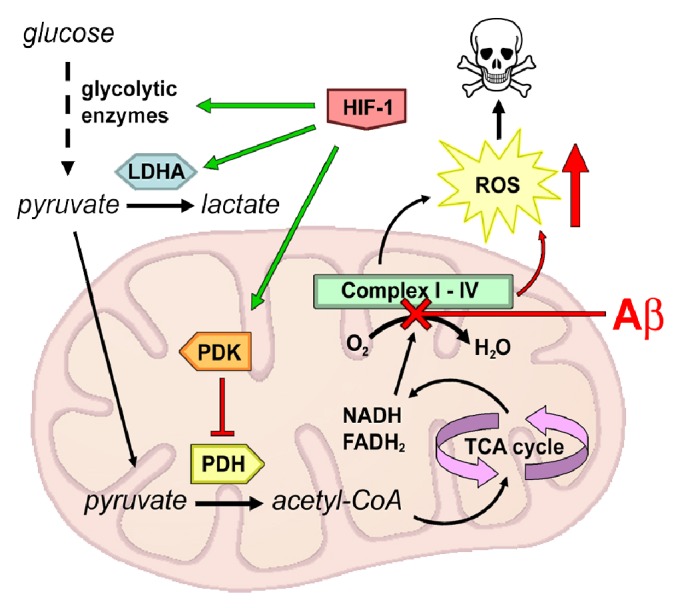
Aerobic glycolysis in A*β*-resistant cells. The stabilization of hypoxia-inducible factor 1 *α* (HIF1*α*) in amyloid beta- (A*β*-) resistant cells stimulates increased expression of glucose transporters and glycolytic enzymes thereby increasing the conversion of glucose to pyruvate. Additionally, HIF-1 induces the transcription of lactate dehydrogenase A (LDHA), resulting in an increase in the conversion of pyruvate to lactate. Furthermore, HIF-1 suppresses mitochondrial respiration by upregulating pyruvate dehydrogenase kinase 1 (PDK1). PDK1 phosphorylates and inhibits pyruvate dehydrogenase (PDH) resulting in decreased flux through the tricarboxylic acid (TCA) cycle and repressed oxidative phosphorylation (OXPHOS). Decreased OXOPHOS attenuates mitochondrial ROS production rendering cells more resistant to apoptosis in the presence of A*β*. In cells failing to undergo aerobic glycolysis, increased mitochondrial respiration potentiates A*β*-mediated ROS production to toxic levels resulting in cell death.

Intriguingly, decreased expression of both LDHA and PDK1 was observed in cortical extracts of 12-month-old AD transgenic (APPswe/PSEN1dE9) mice [97]. A loss of PDK1 expression was also observed in postmortem cortical tissue from AD patients. It should be noted that this study did not distinguish between expression of PDK1 in nerve and glial cells. Immunohistochemical analysis of PDK1 and LDHA in surviving neurons and astrocytes in AD brain would offer further insight into the relationship of these proteins in protecting against A*β* toxicity. Interestingly, the activity of LDHA has been previously shown to be elevated in the frontal and temporal cortex of patients with AD [99]. In contrast, a reduction in both expression and activity of PDH, the molecular target of PDK1, has been reported in the AD brain [86, 100–102]. However, the activity of PDK1 in AD brain tissue has not been examined. Collectively, these findings indicate that PDK1- or LDHA-mediated aerobic glycolysis protects against A*β* toxicity while maintaining cellular ATP levels (Figure 2). Loss of these proteins may contribute to the cognitive decline and nerve cell death observed in AD.

## 8. Aerobic Glycolysis, AD, and Cancer

The progressive decline in cerebral glucose utilization is known to occur with age and in AD, possibly contributing to both nerve cell loss and memory decline [103, 104]. Although a reduction in cerebral glucose metabolism, as measured by FDG-PET, is commonly used in the diagnosis of AD, recent evidence suggest that glucose utilization is more complex in the AD brain [105, 106]. A recent PET imaging study, which measured both glucose consumption and oxygen utilization, revealed a strong correlation between the spatial distribution of elevated aerobic glycolysis and A*β* plaques in brain tissue from patients with AD, as well as normal individuals with high levels of A*β* deposition but without clinical manifestation of the disease [106]. In the developing nervous system, aerobic glycolysis is believed to account for 90% of glucose consumed [107]. During childhood this fraction accounts for 35% of glucose utilization and finally drops to 10–12% in the adult brain [8]. PET studies of cognitively normal individuals have shown an age-associated decrease in FDG uptake in regions of the brain frequently affected in AD, although these studies did not determine what proportion of glucose was processed by aerobic glycolysis versus oxidative phosphorylation [108]. Moreover, recent imaging analysis of patients with AD revealed regional variations in atrophy, hypometabolism, and A*β* deposition [109]. Importantly, A*β* deposition was not significantly related to either atrophy or hypometabolism, prompting the authors to speculate that the observed disconnect between hypometabolism and A*β* levels likely reflects involvement of region-specific pathological or protective mechanisms [109]. In fact, a recent PET imaging analysis revealed that inheritance of the apolipoprotein E4 (ApoE4) allele, a major genetic risk factor for AD, and not A*β* deposition, strongly correlates with reduced cerebral glucose metabolism in normal aging [110]. ApoE4 is a neurotoxic protein that either increases the production of or interferes with clearance of A*β* [111]. However, ApoE4 can promote neurotoxicity in an A*β*-independent manner. In particular, ApoE4 is susceptible to neuron-specific proteolysis which generates C-terminal truncated versions of ApoE4 which localize to mitochondria, bind electron transport chain enzymes, reduce respiration, and cause neurotoxicity [112, 113]. Thus, mitochondrial accumulation of ApoE4 fragments in neurons may be responsible for the hypometabolism observed in AD. In contrast, elevated aerobic glycolysis may arise in certain areas of the brain most susceptible to insult as a preemptive protective mechanism or in response to A*β* accumulation during aging. Loss of this protective mechanism may render certain areas of the brain susceptible to A*β*-induced neurotoxicity.

Interestingly, in a recent study it was found that cancer survivors have a lower risk of developing AD than those without cancer [114]. In contrast, patients who suffered from AD had a lower risk of incident cancer [114]. It is possible that individuals with cancer also have a higher propensity to activate aerobic glycolysis, as this form of metabolism confers a growth and survival advantage (i.e., antiapoptotic function) to cancer cells [96]. However, individuals who survive cancer may still have higher innate levels of aerobic glycolysis, presumably in areas of the brain, which may protect against the development of AD. In contrast, patients with AD may have lower levels of aerobic glycolysis, which not only renders them susceptible to the toxic effects of A*β* but also leads to decreased susceptibility to developing cancer. A similar trend has been observed in Parkinson's disease [115, 116]. Thus it may be valuable in the future to examine aerobic glycolysis in a Parkinson's disease context. Taken together these data suggest an inverse association between cancer and the development of neurodegenerative diseases. Future studies that examine the direct relationship between aerobic glycolysis, AD, and cancer may provide more insight into this fascinating inverse relationship.

## 9. Lactate Is a Neuroprotective Metabolite

Although aerobic glycolysis and associated lactate production has been shown to enhance memory, the effect of this metabolism on age-dependent or AD-related memory decline and neuronal loss has never formally been examined. Interestingly, L-lactate treatment following an ischemic insult is neuroprotective and attenuates neurological deficits in mice [117, 118]. Intracerebroventricular or intravenous injection of lactate has also been shown to exert a neuroprotective effect during experimentally induced hypoglycemia or cerebral ischemia [117–119]. Lactate also exerts neuroprotective effects via transcriptional activation of brain-derived neurotrophic factor (BDNF) expression in human astrocytes and the SH-SY5Y cell line [120]. BDNF is a necessary factor for the survival of nerve cells within the CNS and is also essential for long-term memory storage [121]. In addition, under normoxic conditions lactate can promote HIF-1 stabilization by inhibiting prolyl hydroxylase 2 activity, the enzyme responsible for HIF-1*α* degradation [122]. Stabilization of HIF-1 increases glycolysis and lactate production, events associated with resistance to A*β* toxicity [90, 91]. Moreover, exogenous lactate has been shown to increase both MCT1 and cytochrome c oxidase (COX) mRNA and protein expression in L6 cells [123]. Thus lactate can elicit a number of events leading to activation of transcription factors involved in its transport and possible processing through the mitochondria. Furthermore, several studies have reported that lactate increases vasodilation [124, 125], and continuous lactate production in the activated brain may serve as a signaling mechanism to increase blood flow and fuel delivery to the brain. Therefore lactate may function as a versatile signaling molecule by both activating neuroprotective metabolism and promoting increased blood flow to certain regions of the brain.

## 10. Exercise-Induced Lactate Production Enhances Memory

During periods of physical exertion such as exercise, systemic lactate levels increase. Under resting conditions the brain releases small amounts of lactate which increases during exercise or hypoxia [126]. During exercise the cerebral uptake of lactate also increases. As such, the brain plays an active role in the clearance of excessive lactate during exercise [126–130]. The oxidation of lactate in the brain may account for as much as 33% of the total energy substrate used by the brain [126]. In contrast, cerebral glucose uptake is reduced by ~25% when cerebral lactate uptake is increased, suggesting that the brain preferentially consumes lactate during exercise [128]. Therefore, it appears that lactate is an important fuel source for brain metabolism both under normal conditions and during exercise. Given the importance of glycogen-derived lactate for long-term memory, it is feasible that exercise may benefit memory and cognitive function. Indeed, a study that examined the effects of a single bout of exercise on motor memory found that subjects that exercised before or after practice of a motor skill displayed significantly better retention of that skill 24 hrs and 7 days after practicing compared to subjects that did not exercise [131]. Interestingly, those that exercised after practice displayed better retention of the motor skill 7 days after training when compared to those that exercised before [131]. These findings suggest a single bout of exercise before or after learning a motor skill can improve long-term retention of that skill. This retention is maximized when exercise is performed after training, or during the consolidation phase of memory [131]. Moreover, regular exercise has not only been shown to have a positive effect on memory retention but also appears to reduce the risk of developing neurodegenerative diseases including AD. Notably, exercise ameliorated memory deficits and A*β* deposition in APP transgenic mice [132]. In addition, a longitudinal study that followed 716 older individuals without dementia over 4 years assessed the link between exercise and AD [133]. The outcome of the study revealed that a higher level of total daily physical activity was associated with a reduced risk of AD [133]. Similar results were obtained when individuals were followed over a 14-year period [134].

Interestingly BNDF is significantly elevated in response to exercise, possibly through increased lactate production, which may also account for some of the neuroprotective effects of exercise [135]. It should be noted that intravenous sodium lactate administration in AD patients failed to improve cognitive functioning, although it did slightly improve semantic memory [136]. However, this study only examined the effects of a single 20 min intravenous administration of sodium lactate on cognitive function. Because lactate was only administered for a short period it is unknown if longer periods or multiple administrations would improve cognitive function in AD patients. The systematic administration of glucose to AD patients does, however, improve memory [137]. In contrast, insulin deficiency in AD transgenic mice exacerbates the AD phenotype [138]. Notably, AD patients are at an increased risk for type II diabetes, indicating an important association between glucose uptake and disease progression [139]. Furthermore, patients with type II diabetes are at increased risk for developing cognitive defects, AD, or related dementias [140, 141]. Collectively, these observations suggest that glucose uptake, aerobic glycolysis, and associated lactate production may play a key role in promoting neuronal survival and preventing memory loss during aging and in AD. However, the role of aerobic glycolysis in maintaining CNS neuronal function during aging and preventing AD progression has never experimentally been examined. Clearly alterations in brain metabolism are tightly linked to AD, and future research should focus on mechanisms that either enhance glucose uptake, aerobic glycolysis, or lactate production.

## 11. Conclusions and Future Studies

In light of recent research, lactate has emerged as an important metabolite in the brain. These new findings have altered the context in which we look at the brain and its functioning. Importantly, given that metabolic dysfunction is tightly linked to neurodegenerative diseases, including AD, further studies measuring aerobic glycolysis *in vivo* are warranted. Considering the relationship between aerobic glycolysis in the AD brain, as well as the observed protective effect of aerobic glycolysis against A*β* *in vitro,* it would be valuable to perform a longitudinal study of aerobic glycolysis in normal and AD patients using FDG-PET to determine if elevated or sustained aerobic glycolysis correlates with better clinical outcome. Recent results from the dominantly inherited Alzheimer's network (DIAN) study showed that A*β* accumulation preceded detectable atrophy and hypometabolism by decades [142, 143]. Interestingly, the caudate nucleus had very high levels of A*β* deposition but did not exhibit hypometabolism or neuronal loss throughout the course of disease progression [143]. Assuming that the caudate nucleus is resistant to the toxic effects of AD it would be valuable to determine if aerobic glycolysis is also elevated in this brain region.

A significant proportion of elderly individuals exhibit sufficient plaque accumulation warranting a neuropathology-based classification as probable AD yet are normal by cognitive assessments [144, 145]. Assuming A*β* accumulation promotes neurotoxicity and dementia, then increased CNS aerobic glycolysis may arise as a protective mechanism to enable these individuals to evade cognitive decline. Examining the brains of nondemented individuals with AD neuropathology (NDAN) may shed light on these neuroprotective mechanisms. Given the protective nature of LDHA and PDK1 *in vitro*, it would be of interest to examine the expression patterns of these enzymes in NDAN individuals. Moreover, transgenic (tg) AD mice also may provide an interesting model for uncovering neuroprotective mechanisms that arise from the constitutive and progressive production of A*β* in the CNS. Although tg-AD mice (APP/PS1) have high plaque loads by 6 months of age, most mice exhibit little to no nerve cell loss and are cognitively indistinguishable from nontransgenic mice, whereas synaptic dysfunction and neurotoxicity occurs in tg-AD mice older than 6 months [146, 147]. Thus young tg-AD mice may offer a model of A*β* resistance. Interestingly, we have observed elevated expression of PDK1 in cortical extracts from young (3-month-old) tg-AD mice, which may be indicative of a compensatory protective mechanism (unpublished observations). Creation of tg mice expressing enzymes that regulate aerobic glycolysis in the CNS, crossed to tg-AD mice, may help further define the role of this metabolism in preventing cognitive decline in older mice. These studies would provide a strong rationale for identifying compounds which activate aerobic glycolysis and enhance CNS function. The wealth of new research demonstrating the importance of aerobic glycolysis in the brain holds promise that activation of this form of metabolism may offer a new therapeutic strategy for the treatment of AD and other neurodegenerative diseases.
